# Association of Childhood Social-Emotional Functioning Profiles at School Entry With Early-Onset Mental Health Conditions

**DOI:** 10.1001/jamanetworkopen.2018.6694

**Published:** 2019-01-04

**Authors:** Kimberly C. Thomson, Chris G. Richardson, Anne M. Gadermann, Scott D. Emerson, Jean Shoveller, Martin Guhn

**Affiliations:** 1 Human Early Learning Partnership, School of Population and Public Health, University of British Columbia, Vancouver, British Columbia, Canada; 2 School of Population and Public Health, University of British Columbia, Vancouver, British Columbia, Canada; 3 Centre for Health Evaluation and Outcome Sciences, St Paul’s Hospital, Vancouver, British Columbia, Canada

## Abstract

**Importance:**

More than 50% of lifetime mental health disorders develop by early adolescence, and yet it is not well understood how early childhood social-emotional functioning varies in populations or how differences in functioning may be associated with emerging mental health conditions.

**Objectives:**

To identify profiles of social-emotional functioning at kindergarten school entry (age 5 years) and to examine to what extent profiles are related to early-onset mental health conditions (ages 6-14 years).

**Design, Setting, and Participants:**

This prospective cohort study followed up a population cohort of 34 552 children in British Columbia, Canada, from birth (born 1996-1998) to age 14 years (last follow-up, December 31, 2011). Data were analyzed from the Developmental Trajectories cohort that links British Columbia child development data from the Early Development Instrument (EDI) to British Columbia Ministry of Health and Ministry of Education records. Data were analyzed between May and September 2017.

**Exposures:**

Early childhood social-emotional functioning (defined as social competence, internalizing, and externalizing symptoms) rated by the children’s kindergarten teachers.

**Main Outcomes and Measures:**

Occurrences of physician-assessed mental health conditions throughout childhood and early adolescence, including depression, anxiety, conduct disorder, and attention-deficit/hyperactivity disorder (ADHD), calculated from billing codes from the *International Classification of Diseases, Ninth Revision* recorded in provincial health insurance data.

**Results:**

Data from 34 323 children (mean [SD] age, 5.7 [0.3] years; 17 538 [51.1%] were boys) were analyzed at kindergarten and followed up to age 14 years (15 204 completed follow-up). Latent profile analysis identified 6 unique social-emotional functioning profiles at school entry, with 41.6% of children (n = 14 262) exhibiting comparative vulnerabilities in internalizing or externalizing behaviors. Prevalence of mental health conditions from ages 6 to 14 years was 4.0% for depression, 7.0% for anxiety, 5.5% for conduct disorder, 7.1% for ADHD, and 5.4% for multiple conditions. Zero-inflated Poisson analyses showed an association between social-emotional functioning profiles at kindergarten school entry and physician-assessed mental health conditions by age 14 years (range of adjusted odds ratios: depression, 1.10 [95% CI, 0.76-1.60] to 2.93 [95% CI, 1.93-4.44]; anxiety, 1.00 [95% CI, 0.74-1.36] to 1.73 [95% CI, 1.11-2.70]; conduct disorder, 2.17 [95% CI, 1.41-3.34] to 6.91 [95% CI, 4.90-9.74]; ADHD, 1.46 [95% CI, 1.11-1.93] to 8.72 [95% CI, 6.46-11.78]; and multiple conditions, 1.20 [95% CI, 0.88-1.63] to 6.81 [95% CI, 4.91-9.44]). Children with higher teacher ratings of aggression and hyperactivity had more frequent consultations for conduct disorder, ADHD, and multiple conditions.

**Conclusions and Relevance:**

This study’s findings suggest that more than 40% of children enter the school system with relative vulnerabilities in social-emotional functioning that are associated with early-onset mental health conditions. The results raise important questions for using population-level early childhood development monitoring in the context of universal and proactive mental health strategies.

## Key Points

**Question:**

What can population patterns of early childhood social-emotional functioning tell us about the emergence of mental health conditions?

**Findings:**

In this cohort study that included 34 323 children in Canada, 6 latent social-emotional functioning profiles based on children’s relative strengths and vulnerabilities in social competence, internalizing, and externalizing symptoms at age 5 years were associated with the onset of subsequent mental health conditions between ages 6 and 14 years.

**Meaning:**

This examination of early childhood social-emotional functioning profiles identified social disparities in profile membership and an association between profiles and the emergence of mental health conditions.

## Teaser

This cohort study identifies 6 unique childhood social-emotional functioning profiles at kindergarten school entry and examines the association of these profiles with early-onset mental health conditions in children 6 to 14 years of age.

## Introduction

Early adolescence is a developmental period when many mental health conditions are first diagnosed, yet more than half of individuals with lifetime mental health problems report first experiencing symptoms before age 14 years.^1,2^ Previous studies identifying childhood social-emotional vulnerabilities as early as preschool (including internalizing and externalizing symptoms) suggest that opportunities to intervene may occur even earlier.^3,4^ However, the early detection and prevention of mental health problems remain poorly addressed in part because of inadequate systems to support the identification of early subclinical indicators and associated interventions before problems reach a clinical stage.^3,5,6^ Efforts to identify and address specific mental health problems are also often impeded by the blurring of “boundaries” between symptoms,^7^ when examining patterns of shared symptoms routinely collected during periods of transition and development may better inform early identification and intervention at a population level. This possibility led us to use latent profile analysis^8,9,10^ to explore multiple indicators of interest (eg, sadness, exploration, and restlessness) as a means of identifying profiles of early childhood social-emotional functioning as children enter the school system that may signal the development of early-onset mental health conditions. In a previous investigation that informed the present study, social-emotional functioning was defined in terms of early childhood psychosocial health, including social competence, internalizing symptoms (inhibition, depressive symptoms, and overcontrolled behaviors), and externalizing symptoms (hyperactivity, aggression, and undercontrolled behaviors).^11^

Common early behavioral indicators associated with increased risk of internalizing and externalizing problems in childhood include heightened fearfulness as well as chronic irritability, fatigue, and difficulty concentrating.^12,13,14^ Sometimes a particular condition will worsen over time (eg, attention-deficit/hyperactivity disorder [ADHD] persisting from early childhood to adolescence)^15^; in other cases, an underlying condition will express itself in different symptoms or behaviors over time.^16,17^ Numerous longitudinal studies have shown that early symptoms of internalizing and externalizing conditions are associated with earlier onset and increased severity of those same conditions later in life, particularly for depression and conduct problems.^18,19,20,21,22^ However, other research has shown a clear sequential progression between early childhood symptoms of a single condition (eg, ADHD) and adolescent onset of a different condition (eg, anxiety),^16,17^ and this progression has been explained by both biological and social mechanisms.^23,24,25^ In the context of childhood social-emotional functioning, key knowledge gaps remain, including how internalizing and externalizing symptoms co-occur in the context of positive behaviors, such as social skills, and the extent to which patterns of early social-emotional symptoms are associated with mental health conditions.

### Objectives

This study had 2 objectives: (1) to identify latent profiles of early social-emotional functioning among a population cohort of children at school entry and (2) to examine the association between these early childhood social-emotional functioning profiles and physician-assessed mental health conditions throughout childhood and early adolescence (including depression, anxiety, conduct disorder, and ADHD). Longitudinal associations between internalizing and externalizing conditions were also examined to explore the extent to which early symptoms continued or changed as children reached adolescence.

## Methods

### Data Source

This study followed the Strengthening the Reporting of Observational Studies in Epidemiology (STROBE) reporting guidelines. Ethics approval was obtained from the University of British Columbia Research Ethics Board. Data were analyzed from the Developmental Trajectories cohort^26^ that links British Columbia child development data from the Early Development Instrument (EDI)^27^ to British Columbia Ministry of Health and Ministry of Education records. Identification of latent profiles at kindergarten entry was based on a population cohort of children born between 1996 and 1998 (N = 34 552). Analyses with mental health outcomes were restricted to a subsample of children with complete Medical Service Plan (MSP) health insurance records from 1996 to 2011 (ie, children registered with an MSP account for ≥9 months of every year for the 14 years following their birth) (n = 20 409). Children included in this cohort had EDI data collected between 2001 and 2003. Within the EDI data collection period, 86% of children attending kindergarten in British Columbia, Canada, were enrolled in public schools and captured within the EDI population sample.^28^ Children included in the linked EDI-MSP subsample included all citizens and permanent residents.^29^ Data were analyzed between May and September 2017.

### Measures

#### Explanatory Variable: Social-Emotional Functioning at School Entry

Eight subscales of British Columbia’s EDI^27,30,31^ database were used as indicators of children’s social-emotional functioning at school entry (EDI social-emotional subscales: overall social competence, responsibility and respect, approaches to learning, readiness to explore, prosocial and helping behavior, anxious and fearful behavior [reverse-coded, ie, so that higher scores indicated better social-emotional functioning for every subscale], aggressive behavior [reverse-coded], and hyperactive and inattentive behavior [reverse-coded]). The EDI is a validated population-level, teacher-report measure of children’s development within a school-based context that has been implemented across Canada and internationally to monitor cross-sectional and longitudinal associations between early childhood development and children’s sociodemographic circumstances, health, and education.^32,33,34,35^ Previous studies have found the EDI to have good interrater reliability (range of correlations between teacher and early childhood educator ratings, 0.53-0.80) and to provide unbiased measurement when comparing teacher ratings by child sex, English as a second language (ESL) status, and Aboriginal status.^27,36^ Example items, means and SDs, and ordinal α values^37^ are provided in Table 1. For each item within the 8 EDI social-emotional subscales, teachers rated their students’ behavior currently or within the past 6 months as “never or not true” (score of 0), “sometimes or somewhat true” (score of 5), or “often or very true” (score of 10) (where higher scores indicate better social-emotional functioning). “Don’t know” was coded as missing. Scores for each item were summed and divided by the number of items in the subscale to derive a subscale mean, and negatively worded items were reverse-coded for the anxious and fearful, aggressive, and hyperactive and inattentive subscales so that higher scores indicated better social-emotional functioning. This EDI scoring was developed in consultation with educators and community audiences without prior clinical or research background for ease of interpretation and dissemination of findings.^27^

**Table 1.** Means, Distributions, and Reliability of the 8 Early Development Instrument (EDI) Social-Emotional Functioning Subscales Used to Assess Children Attending Kindergarten in British Columbia, Canada

EDI Subscale	Subscale Items	Unstandardized, Mean Score (SD)^a^	Ordinal α
Overall social competence	Overall social/emotional development Ability to get along with peers Plays and works cooperatively with other children at the level appropriate for his/her age Is able to play with various children Shows self-confidence	7.59 (2.51)	.92
Responsibility and respect	Follows rules and instructions Respects the property of others Demonstrates self-control Demonstrates respect for adults Demonstrates respect for other children Accepts responsibility for actions Takes care of school materials Shows tolerance to someone who made a mistake (eg, when a child gives a wrong answer to a question posed by the teacher)	8.55 (2.02)	.97
Approaches to learning	Listens attentively Follows directions Completes work on time Works independently Works neatly and carefully Is able to solve day-to-day problems by himself/herself Is able to follow 1-step instructions Is able to follow class routines without reminders Is able to adjust to changes in routines	8.01 (2.18)	.95
Readiness to explore	Is curious about the world Is eager to play with a new toy Is eager to play a new game Is eager to play with/read a new book	8.94 (1.90)	.95
Prosocial and helping behavior	Will try to help someone who has been hurt Volunteers to help clear up a mess someone else has made If there is a quarrel or dispute, will try to stop it Offers to help other children who have difficulty with a task Comforts a child who is crying or upset Spontaneously helps to pick up objects that another child has dropped (eg, pencils, books) Will invite bystanders to join in a game Helps other children who are feeling sick	5.72 (2.98)	.96
Anxious and fearful	Is upset when left by parent or guardian Seems to be unhappy, sad, or depressed Appears fearful or anxious Appears worried Cries a lot Is nervous, high-strung, or tense Is incapable of making decisions Is shy	8.90 (1.57)	.91
Aggressive behavior	Gets into physical fights Bullies or is mean to others Kicks, bites, hits other children or adults Takes things that do not belong to him or her Laughs at other children’s discomfort Is disobedient Has temper tantrums	9.23 (1.48)	.94
Hyperactive and inattentive	Cannot sit still, is restless Is distractible, has trouble sticking to any activity Fidgets Is impulsive, acts without thinking Has difficulty awaiting turn in games or groups Cannot settle to anything for more than a few moments Is inattentive	8.16 (2.46)	.97

^a^ Subscales range from 0 to 10, with higher scores indicating better social-emotional functioning.

#### Covariates

Covariates were selected to control for potential confounding related to child age, sex, and first language as well as family sociodemographic factors (household income, parent marital status at child birth, and maternal age at child birth) that could account for differences in observed child behaviors, health service use, and mental health diagnoses.^38,39,40,41,42,43,44^ Child age, sex (male or female), and ESL status were derived from teachers’ ratings and cross-validated with British Columbia Ministry of Education records. Parent marital status and mother’s age were taken from MSP records collected at the time of the child’s birth. Marital status was dichotomized into “married” and “not married” (never married, divorced, separated, widowed, other), whereas maternal age was divided into 3 categories (<20, 20-35, and >35 years) because younger and older maternal age has been shown to be associated with developmental vulnerabilities in children.^44^ Household subsidy status from MSP records was used as an indicator of relative poverty, with “100% subsidy” indicating socioeconomic disadvantage (during the study period, full subsidies were available to a family of any size that earned an annual net income <$22 000 Can $) compared with families earning more than $30 000 who qualified for “no subsidy.” Ten percent of households did not fall into these 2 categories and were recorded as having missing data on this variable.^11^

#### Outcome Variable: Mental Health Conditions

Mental health conditions were obtained from physician claims files that were recorded in the MSP data for anxiety, depression, conduct disorder, and ADHD. A fifth outcome, multiple conditions, was derived that included children who had received 2 or more of the above diagnoses during the study period. Notably, although multiple conditions may have been comorbid, it is also possible that they presented at different times in development. Within the available MSP records, medical services provided were identified using the *International Classification of Diseases, Ninth Revision* (*ICD-9*)^45^: anxiety disorder (parent code, 300), depression (codes, 296.2-296.36; parent code, 311), conduct disorder (parent code, 312), and ADHD (parent code, 314). Every physician visit that included consultation (and a consequent corresponding billing claim) for a mental health condition was coded as an occurrence of a mental health condition; therefore, children’s health records could accumulate multiple occurrences for the same mental health condition over the study period. Only records incurred at 6 years or older were included in the analyses to assess the prospective association between latent profiles at school entry and subsequent mental health conditions.

### Statistical Analysis

#### Identifying Latent Social-Emotional Functioning Profiles

Latent profile analysis was performed using MPlus, version 8 (Muthén & Muthén),^46^ to identify profiles of social-emotional functioning. This analysis compares nested models to assess how well profiles of grouped underlying latent classes explain the variance among a set of predictor variables.^8,9,10^

In this study, predictor variables were the 8 social-emotional EDI subscale scores, *z*-standardized within the sample (mean [SD], 0 [1]). Children who had missing data on all 8 social-emotional subscales were excluded. The best overall model solution was identified based on multiple starting values.^47,48^ Model fit was assessed according to (1) entropy score closest to 1, indicating good classification accuracy; (2) high discrimination between classification probabilities (probability of being assigned to any particular class ≥0.8); (3) lowest adjusted Bayesian information criterion (aBIC), indicating relatively better fit among nested models; and (4) statistically significant Bayesian likelihood ratio test, testing whether a model with k latent classes fits better than a model with k-1 classes.^47,49^ Parsimony, interpretability, and theoretical meaningfulness were also considered in the interpretation of the best model.^47,49^ In the final step, each child was assigned a latent profile membership value based on his or her most likely profile membership.

#### Association Between Latent Social-Emotional Profiles and Mental Health Conditions

Zero-inflated Poisson (ZIP) regression analysis was conducted to assess the association between children’s kindergarten social-emotional functioning profiles and the occurrence of mental health visits for anxiety, depression, conduct disorder, ADHD, and multiple conditions. Zero-inflated Poisson (ZIP) models are often used in studies in which the occurrence of the outcome is rare (ie, in count data with a high preponderance of zeroes), including studies examining clinical mental health diagnoses in childhood and adolescence.^10,50,51,52^ The first part of the ZIP model calculates a dichotomous latent outcome: the odds of children belonging to a class that always scores 0 on the outcome (always 0 class) vs a class for which 1 or more mental health conditions are possible (not always 0 class). The second part of the model estimates the frequency of events among children in the not always 0 class.^50,53^ *P* values and CIs are provided as part of the ZIP regression analysis. In the present study, statistical significance was assessed at α less than .05 (2-sided).

## Results

### Latent Profile Analysis

Among 34 552 children included in the initial Developmental Trajectories data set, 229 children (0.7%) were excluded for having missing data on all 8 EDI social-emotional subscales. The remaining 34 323 children (mean [SD] age at kindergarten entry, 5.67 [0.30] years; range, 4.41-7.18 years) were included in the latent profile analysis analytic sample. Within this cohort of children, 51.1% (17 538) were identified as boys, 48.9% (16 785) were identified as girls, 15.1% (5098) had ESL status, 18.4% (5571) were from households that had received a full subsidy at the earliest health care visit, and 26.9% (8019) had parents who were unmarried. At the time of the child’s birth, 4.8% of mothers (1483) were younger than 20 years, and 11.9% (3652) were older than 35 years.

Compared with the other solutions, the 6-class model showed improved model fit over models with fewer classes (ie, statistically significant Bayesian likelihood ratio test and lower log-likelihood and aBIC scores) while still demonstrating high entropy (94%) and high probability of children of being assigned to a specific class (90%). Model fit comparisons are available in eTable1 in the Supplement. Log-likelihood and aBIC scores continued to decrease in models with 7 to 10 latent classes; however, these decreases were relatively small compared with the differences between models with 1 to 6 latent classes. Based on these diminished improvements in later models and subsequent losses in entropy and class probability accuracy, the 6-class solution was determined to fit the data best.^49^ A sensitivity analysis conducted on the smaller cohort of children with complete follow-up included in the subsequent ZIP analysis (n = 15 204) replicated these results.

The 6 social-emotional functioning profiles are presented in the Figure (profile means and SDs are available in eTable 2 in the Supplement). Most children had high scores across all 8 EDI subscales (profile 1, 58.4% [20 061]). The remaining 41.6% of children (14 262) were classified into profiles depicting different patterns of vulnerability in 1 or more social-emotional domain(s) (profile 2, 8.3% [2856]; profile 3, 16.4% [5622]; profile 4, 6.2% [2144]; profile 5, 7.8% [2677]; and profile 6, 2.8% [963]). Sociodemographic comparisons showed that boys, children from households with unmarried parents, younger mothers, and households receiving subsidies were increasingly overrepresented in each more vulnerable social-emotional functioning profile (Table 2), and children with ESL status were overrepresented in the inhibited profiles (profiles 2 and 4).

**Figure.**
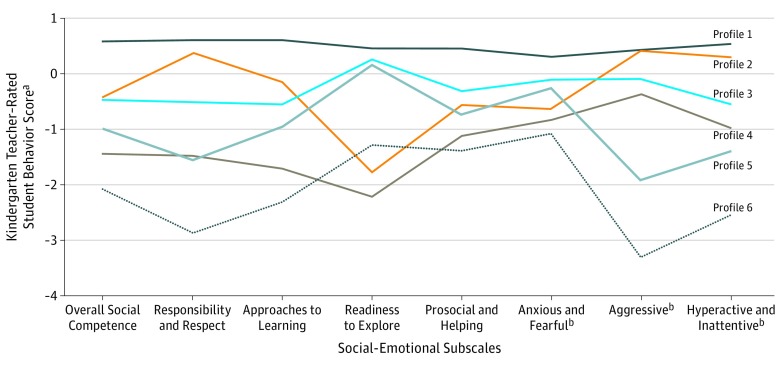
Composition of the 6 Latent Social-Emotional Functioning Profile Groups by 8 Early Development Instrument (EDI) Social-Emotional Subscales and Population Prevalence Among Children Attending Kindergarten in British Columbia, Canada Social-emotional functioning profiles (and population prevalence): 1, overall high social-emotional functioning (58.4%); 2, inhibited-adaptive (8.3%); 3, uninhibited-adaptive (16.4%); 4, inhibited-disengaged (6.2%); 5, uninhibited-aggressive and hyperactive (7.8%); and 6, overall low social-emotional functioning (2.8%). ^a^For each item, teachers rated a student’s behavior currently or within the past 6 months as “never or not true” (score of 0), “sometimes or somewhat true” (score 5), or “often or very true” (score of 10). “Don’t know” was coded as missing. Scores for each subscale item were summed and divided by the number of items in the subscale to derive a subscale mean that was then z-standardized within the sample (indicated by the horizontal line at 0). Negatively worded items were reverse-coded for the anxious and fearful, aggressive, and hyperactive and inattentive subscales so that higher scores indicated better social-emotional functioning (used for the other positively worded subscales). This EDI scoring was developed in consultation with educators and community audiences without prior clinical or research background for ease of interpretation and dissemination of findings. ^b^Reverse coded.

**Table 2.** Sociodemographic Characteristics of 34 323 Children by Latent Social-Emotional Functioning Profile Group

Sociodemographic Characteristic	Latent Social-Emotional Functioning Profile Group, No. (%) of Children^a^					
	Profile 1	Profile 2	Profile 3	Profile 4	Profile 5	Profile 6
Total sample	20 061 (58.4)	2856 (8.3)	5622 (16.4)	2144 (6.2)	2677 (7.8)	963 (2.8)
Boys	8685 (43.3)	1272 (44.5)	3481 (61.9)	1349 (62.9)	1957 (73.1)	794 (82.5)
Girls	11 376 (56.7)	1584 (55.5)	2141 (38.1)	795 (37.1)	720 (26.9)	169 (17.5)
ESL status	2616 (13.2)	578 (20.6)	879 (15.9)	532 (25.2)	357 (13.5)	136 (14.4)
Receiving subsidies	2755 (15.2)	511 (20.5)	1034 (21.2)	491 (27.3)	545 (24.2)	235 (31.6)
Unmarried parents	3958 (22.5)	678 (27.2)	1519 (31.2)	644 (35.6)	851 (37.8)	369 (46.5)
Maternal age, mean (SD), y	29.3 (5.4)	29.0 (5.5)	28.6 (5.7)	28.1 (5.9)	28.0 (6.0)	27.7 (6.3)
Child age, mean (SD), y	5.7 (0.3)	5.6 (0.3)	5.6 (0.3)	5.6 (0.3)	5.7 (0.3)	5.7 (0.4)

Abbreviation: ESL, English as a second language.

^a^ Percentages are based on valid percentage within the sample. Latent social-emotional functioning profiles: 1, overall high social-emotional functioning; 2, inhibited-adaptive; 3, uninhibited-adaptive; 4, inhibited-disengaged; 5, uninhibited-aggressive and hyperactive; and 6, overall low social-emotional functioning.

### ZIP Models

The ZIP analysis was conducted with the longitudinal subsample of 20 409 children for whom we had MSP data from birth to age 14 years (complete follow-up). Of these, 5205 were excluded from the analyses owing to missing data (n = 3681) or the occurrence of a mental health condition before or concurrent with the age at which EDI data were collected (n = 1524). The final ZIP cohort included 15 204 children. Children not included in the ZIP analysis were more likely to come from households receiving subsidies (21.5% of children not in the ZIP analysis vs 15.3% of children in the ZIP analysis; χ^2^_1_ = 195.74; *P* < .001), have unmarried parents (35.1% of children not in the ZIP analysis vs 19.0% of children in the ZIP analysis; χ^2^_1_ = 985.95; *P* < .001), be born to younger mothers (mean [SD] maternal age at birth: 28.4 [5.8] years for children not in the ZIP analysis vs 29.6 [5.2] years for children in the ZIP analysis; *t*_30,656_ = 19.25; *P* < .001), and to have ESL status (18.8% of children not in the ZIP analysis vs 10.6% of children in the ZIP analysis; χ^2^_1_ = 443.55; *P* < .001). No differences were found regarding child age or sex.

Five ZIP models, associated with each mental health outcome separately, identified a consistent gradient pattern for all mental health outcomes. Adjusted odds ratios (aORs) and 95% CIs are presented in Table 3. Adjusted rate ratios (aRRs) and 95% CIs (assessing the frequency of mental health consultations) are presented in Table 4. Overall, ESL status was associated with lower odds of a mental health condition (range of aORs: depression, 0.60 [95% CI, 0.39-0.93] to multiple conditions, 0.43 [95% CI, 0.31-0.59]), and boys had higher odds of externalizing conditions (aORs: conduct disorder, 1.67 [95% CI, 1.39-2.00] and ADHD, 2.08 [95% CI, 1.80-2.40]. Household factors (receiving subsidies, unmarried parent, and younger mother) were associated with higher odds of a mental health condition, but these associations were generally not statistically significant (range of aORs from depression to multiple conditions: receiving subsidy, 1.05 [95% CI, 0.78-1.42] to 1.06 [95% CI, 0.88-1.28]; unmarried parent, 1.20 [95% CI, 0.94-1.53] to 1.49 [95% CI, 1.25-1.77]; and younger mother, 1.39 [95% CI, 0.81-2.38] to 1.21 [95% CI, 0.86-1.70]). After adjustment for demographic characteristics, children classified in successively more vulnerable social-emotional functioning profiles in kindergarten (profiles 2-6 vs profile 1) generally had incrementally higher odds of a physician-assessed mental health diagnosis of depression, anxiety, conduct disorder, ADHD, and 2 or more (multiple) conditions from ages 6 to 14 years (range of aORs: depression, 1.10 [95% CI, 0.76-1.60] for profile 2 to 2.93 [95% CI, 1.93-4.44] for profile 4; anxiety, 1.00 [95% CI, 0.74-1.36] for profile 2 to 1.73 [95% CI, 1.11-2.70] for profile 6; conduct disorder, 2.17 [95% CI, 1.41-3.34] for profile 2 to 6.91 [95% CI, 4.90-9.74] for profile 6; ADHD, 1.46 [95% CI, 1.11-1.93] for profile 2 to 8.72 [95% CI, 6.46-11.78] for profile 6; multiple conditions, 1.20 [95% CI, 0.88-1.63] for profile 2 to 6.81 [95% CI, 4.91-9.44] for profile 6). The pattern of results for the frequency of mental health consultations was not as consistent: For conduct disorder, ADHD, and multiple conditions, children with the highest aggression and hyperactivity (profiles 5 and 6) had a higher number of consultations than children with overall high social-emotional functioning (profile 1); however, this pattern was not observed for depression or anxiety.

**Table 3.** Summary of Zero-Inflated Poisson Results by Occurrence of Mental Health Condition: Adjusted Odds Ratios Among 15 204 Children Attending Kindergarten in British Columbia, Canada, From Ages 6 to 14 Years

Explanatory Variable	Adjusted Odds Ratio (95% CI) by Prevalence of Condition at Follow-up				
	Depression (4.0% at Follow-up)	Anxiety (7.0% at Follow-up)	Conduct Disorder (5.5% at Follow-up)	Attention-Deficit/Hyperactivity Disorder (7.1% at Follow-up)	Multiple Conditions (5.4% at Follow-up)
Child age^a^	1.25 (0.88-1.77)	1.01 (0.79-1.29)	0.90 (0.69-1.18)	0.79 (0.64-0.98)^b^	0.86 (0.68-1.09)
Sex					
Girl	1 [Reference]	1 [Reference]	1 [Reference]	1 [Reference]	1 [Reference]
Boy	0.82 (0.67-1.01)	0.98 (0.84-1.14)	1.67 (1.39-2.00)^c^	2.08 (1.80-2.40)^c^	1.45 (1.24-1.69)^c^
English language status					
English as first language	1 [Reference]	1 [Reference]	1 [Reference]	1 [Reference]	1 [Reference]
English as second language	0.60 (0.39-0.93)^d^	0.67 (0.50-0.89)^b^	0.70 (0.46-1.06)	0.39 (0.29-0.52)^c^	0.43 (0.31-0.59)^c^
Household subsidy status					
Not receiving subsidy	1 [Reference]	1 [Reference]	1 [Reference]	1 [Reference]	1 [Reference]
Receiving subsidy	1.05 (0.78-1.42)	0.98 (0.78-1.23)	0.94 (0.76-1.16)	1.08 (0.91-1.29)	1.06 (0.88-1.28)
Maternal age at child birth, y					
<20	1.39 (0.81-2.38)	1.48 (0.79-2.76)	1.23 (0.84-1.79)	1.25 (0.91-1.71)	1.21 (0.86-1.70)
20-35	1 [Reference]	1 [Reference]	1 [Reference]	1 [Reference]	1 [Reference]
>35	0.99 (0.74-1.32)	1.22 (0.99-1.50)	1.07 (0.83-1.36)	1.08 (0.89-1.31)	1.31 (1.07-1.61)^d^
Parent marital status at birth					
Married	1 [Reference]	1 [Reference]	1 [Reference]	1 [Reference]	1 [Reference]
Unmarried	1.20 (0.94-1.53)	1.19 (0.98-1.45)	1.43 (1.18-1.74)^c^	1.25 (1.05-1.47)^c^	1.49 (1.25-1.77)^c^
Latent social-emotional functioning profile group^e^					
Profile 1	1 [Reference]	1 [Reference]	1 [Reference]	1 [Reference]	1 [Reference]
Profile 2	1.10 (0.76-1.60)	1.00 (0.74-1.36)	2.17 (1.41-3.34)^c^	1.46 (1.11-1.93)^c^	1.20 (0.88-1.63)
Profile 3	1.26 (0.94-1.68)	1.10 (0.88-1.37)	2.38 (1.91-2.97)^c^	3.36 (2.84-3.98)^c^	2.36 (1.95-2.86)^c^
Profile 4	2.93 (1.93-4.44)^c^	1.28 (0.95-1.71)	3.93 (3.00-5.17)^c^	4.25 (3.37-5.36)^c^	3.51 (2.72-4.52)^c^
Profile 5	2.34 (1.66-3.31)^c^	1.35 (1.04-1.75)^d^	3.37 (2.65-4.30)^c^	5.40 (4.43-6.58)^c^	3.89 (3.11-4.86)^c^
Profile 6	1.43 (0.78-2.62)	1.73 (1.11-2.70)^d^	6.91 (4.90-9.74)^c^	8.72 (6.46-11.78)^c^	6.81 (4.91-9.44)^c^

^a^ Child age was measured as a continuous variable and can be interpreted as the change in odds associated with every 1-year increase in age.

^b^ *P* < .05.

^c^ *P* < .001.

^d^ *P* < .01.

^e^ Latent social-emotional functioning profiles: 1, overall high social-emotional functioning; 2, inhibited-adaptive; 3, uninhibited-adaptive; 4, inhibited-disengaged; 5, uninhibited-aggressive and hyperactive; and 6, overall low social-emotional functioning.

**Table 4.** Summary of Zero-Inflated Poisson Results by Frequency of Mental Health Condition: Adjusted Rate Ratios Among 15 204 Children Attending Kindergarten in British Columbia, Canada, From Ages 6 to 14 Years

Explanatory Variable	Adjusted Rate Ratio (95% CI) by Prevalence of Condition at Follow-up				
	Depression (4.0% at Follow-up)	Anxiety (7.0% at Follow-up)	Conduct Disorder (5.5% at Follow-up)	Attention-Deficit/Hyperactivity Disorder (7.1% at Follow-up)	Multiple Conditions (5.4% at Follow-up)
Child age^a^	0.97 (0.54-1.73)	1.37 (0.96-1.96)	0.98 (0.65-1.48)	1.22 (0.97-1.52)	1.30 (1.07-1.58)^b^
Sex					
Girl	1 [Reference]	1 [Reference]	1 [Reference]	1 [Reference]	1 [Reference]
Boy	1.19 (0.89-1.60)	0.89 (0.70-1.14)	0.91 (0.68-1.21)	0.95 (0.80-1.12)	0.95 (0.82-1.09)
English language status					
English as first language	1 [Reference]	1 [Reference]	1 [Reference]	1 [Reference]	1 [Reference]
English as second language	0.83 (0.46-1.47)	0.87 (0.58-1.31)	0.66 (0.34-1.29)	0.70 (0.52-0.93)^c^	0.74 (0.58-0.94)^b^
Household subsidy status					
Not receiving subsidy	1 [Reference]	1 [Reference]	1 [Reference]	1 [Reference]	1 [Reference]
Receiving subsidy	0.85 (0.53-1.37)	0.84 (0.60-1.18)	1.23 (0.88-1.72)	1.12 (0.91-1.36)	1.04 (0.86-1.26)
Maternal age at child birth, y					
<20	0.83 (0.37-1.87)	0.43 (0.22-0.87)^c^	0.84 (0.51-1.37)	0.98 (0.73-1.33)	0.80 (0.62-1.04)
20-35	1 [Reference]	1 [Reference]	1 [Reference]	1 [Reference]	1 [Reference]
>35	1.01 (0.68-1.50)	0.97 (0.71-1.33)	0.89 (0.64-1.22)	1.09 (0.88-1.35)	0.96 (0.80-1.15)
Parents marital status at birth					
Married	1 [Reference]	1 [Reference]	1 [Reference]	1 [Reference]	1 [Reference]
Unmarried	1.26 (0.90-1.78)	0.93 (0.69-1.25)	1.12 (0.84-1.48)	0.96 (0.81-1.15)	1.09 (0.93-1.28)
Latent social-emotional functioning profile group^d^					
Profile 1	1 [Reference]	1 [Reference]	1 [Reference]	1 [Reference]	1 [Reference]
Profile 2	0.93 (0.52-1.64)	1.06 (0.60-1.85)	0.51 (0.30-0.85)^c^	0.78 (0.55-1.09)	1.03 (0.77-1.38)
Profile 3	0.84 (0.55-1.28)	0.96 (0.68-1.35)	1.23 (0.85-1.78)	1.18 (0.97-1.44)	1.23 (1.02-1.47)^c^
Profile 4	0.71 (0.40-1.28)	1.21 (0.83-1.77)	1.20 (0.84-1.71)	1.19 (0.93-1.52)	1.26 (1.03-1.54)^c^
Profile 5	0.78 (0.50-1.22)	1.37 (0.91-2.08)	1.74 (1.23-2.45)^e^	1.51 (1.23-1.86)^e^	1.62 (1.34-1.97)^e^
Profile 6	1.47 (0.65-3.31)	1.22 (0.66-2.25)	1.57 (1.04-2.39)^c^	1.71 (1.31-2.24)^e^	1.73 (1.37-2.18)^e^

^a^ Child age was measured as a continuous variable and can be interpreted as the change in odds associated with every 1-year increase in age.

^b^ *P* < .01.

^c^ *P* < .05.

^d^ Latent social-emotional functioning profiles: 1, overall high social-emotional functioning; 2, inhibited-adaptive; 3, uninhibited-adaptive; 4, inhibited-disengaged; 5, uninhibited-aggressive and hyperactive; and 6, overall low social-emotional functioning.

^e^ *P* < .001.

## Discussion

The objectives of this study were to identify latent profiles of children’s social-emotional functioning at school entry and to assess their association with subsequent mental health conditions incurred by age 14 years. A latent profile analysis identified 6 profiles of children’s early social-emotional functioning, with approximately 10% of children exhibiting relatively high hyperactivity and aggression and approximately 3% exhibiting high comorbid internalizing and externalizing symptoms. Boys were overrepresented in more vulnerable profiles and had higher odds of externalizing conditions by adolescence. Children with ESL status were overrepresented in internalizing profiles, which is in line with a previous study using EDI data that also found that children with ESL status were rated as more inhibited and shy, arguably owing to language barriers and unfamiliarity with cultural scripts.^54^ However, children with ESL status had lower odds of a mental health condition by early adolescence, possibly reflecting barriers and a lower use of health services among immigrant families and thus underestimating the outcome in this subpopulation.^55^

After adjustment for child age, sex, ESL status, and household sociodemographic factors, children exhibiting worse social-emotional functioning at school entry had higher odds of a subsequent mental health condition by age 14 years. Overall, results indicated patterns of progression between early internalizing and externalizing symptoms and later internalizing and externalizing conditions. For example, children who exhibited higher internalizing (high inhibition and anxious behavior) at school entry (profile 4) had higher odds of conduct disorder and ADHD by early adolescence in addition to higher internalizing conditions. Children with higher externalizing (aggression and hyperactivity) at school entry (profile 5) had higher odds of depression and anxiety in addition to externalizing conditions. Among the 3 most vulnerable profile groups, internalizing symptoms in profile 4 (inhibited-disengaged) were not associated with higher odds of an anxiety diagnosis. Rather, it was profile 6, characterized by anxious behavior combined with low social competence, high aggression, and hyperactivity that had the largest magnitude of association with anxiety. These results are consistent with other studies showing progressions from one condition to the next (eg, from preschool conduct problems to school-age depression^18,56^ or childhood ADHD symptoms to adult anxiety^16,17^). Recent research indicates that mental health conditions may have shared genetic and environmental causes and that the expressions of early emotional problems may operate more like a phenotype, with variations in outcomes influenced by children's social, biological, and physical environments.^57,58^ Future research is needed to better understand the mechanisms that contribute to shared mental health symptoms in childhood, with the goal of identifying where progressions from early symptoms to qualified mental health disorders can be interrupted or shifted to less severe conditions.

Patterns of symptom continuity were also observed in that children in profile 4 (inhibited-disengaged) had higher odds of depression by early adolescence, and children in profiles 5 and 6 (characterized by higher externalizing symptoms) had higher odds of conduct disorder and ADHD.^18,19,20,21,22,59^ However, symptom continuity was not observed for children in profile 2 (inhibited-adaptive), who exhibited similarly high internalizing at school entry to children in profile 4, but who also showed high social competence and low aggression and hyperactivity. These results suggest that children's social skills may buffer against later internalizing problems (possibly through peer acceptance and social support)^37,60,61^ or, alternatively, that children with internalizing symptoms who function well socially and academically may be overlooked for identification and treatment.

In general, early social-emotional profiles were not consistently associated with medical consultation rates; however, children with incrementally higher aggression and hyperactivity (profiles 5 and 6) showed a higher number of consultations for conduct disorder, ADHD, and multiple conditions. None of the 6 profile patterns were associated with rates of depression consultations, and this finding may be attributable to the comparatively late age at onset for depressive disorders, which has a median onset age of 30 years.^2^

### Strengths and Limitations

This study used a population-level, linked administrative data set to investigate prospective longitudinal patterns of children’s social-emotional development from birth to age 14 years. The EDI data were collected for the entire population of public school kindergarten children in British Columbia, thus allowing a unique opportunity to investigate patterns among population subgroups without the problems of underrepresenting very high- or low-income families, which can sometimes result from the use of stratified or cluster sampling techniques.^62^

This study had several limitations. Public health insurance data likely underestimated the prevalence of mental health conditions because studies suggest that only 30% of children with mental health concerns are connected to appropriate services.^40^ This may be owing to adults (parents, educators, and physicians) not recognizing mental health problems in young people^6^ and hesitance among young people to seek help because of a similar lack of symptom recognition or because of fear of stigma and social exclusion.^63,64^ Furthermore, not all health service uses in British Columbia are captured by the universal public health insurance system. Family physicians and walk-in clinics (as a common first point of contact) as well as many mental health professionals, including pediatricians, psychiatrists, and psychologists, are covered by public health insurance.^65^ Therefore, because a referral is typically required from a general practitioner to access privatized specialized services, it is unlikely that this limitation affected the odds of the outcome (condition or no condition).^66^ However, this data set may have missed an estimated 12% to 20% of services not covered by MSP resulting in underestimated consultation rates.^67,68^

Another study limitation is the possible misclassification of mental health conditions. Physician claims files have been used for case identification in other Canadian research and generally show good accuracy when compared with survey and hospital outpatient data,^69^ and calculations of mental health outcomes based on billing codes have been found to be congruent with self-reported and diagnostic rates.^70,71^ However, because the current study counted a single recorded diagnosis as evidence of a mental health disorder (as precedential in past research^72,73^), the use of less strict criteria may have resulted in increased false-positives for mental health conditions, potentially biasing associations with profile membership toward the null hypothesis. Similarly, because MSP records were only available in the linked data set until age 14 years, these results underestimated the prevalence of lifetime mental health disorders (particularly depression), again reducing the ability to detect a true association.

## Conclusions

Early mental health indicators are difficult to identify and distinguish, undermining efforts for early detection and intervention.^5,6,7^ This study’s findings show how routinely collected early childhood data can be used to monitor population patterns in childhood mental health to inform further research into interventions that target modifiable factors before mental health conditions become fully developed.^5^ The observed prevalence and inequity of young children’s social-emotional vulnerabilities furthermore illuminate that addressing childhood mental health requires population-level interventions that start early, with the school system being a key point of preventative intervention, as demonstrated in other studies.^74,75^ Our results further support the notion that addressing early social-emotional symptoms, such as aggression or low social competence, may have overlapping benefits for reducing future externalizing disorders as well as internalizing disorders,^16^ and future studies should assess the extent to which this is effective. To have the greatest impact, future research should continue to identify factors within children’s social and structural environments that can be leveraged to promote children’s social-emotional development and mental health from the earliest possible opportunities.

## Notes

Supplement. **eTable 1.** Latent Profile Analysis Model Fit Comparison: Social-Emotional Functioning Among Kindergarten Children in British Columbia, Canada **eTable 2.** Standardized Mean Scores (and SDs) for Early Development Instrument Social-Emotional Subscales by Latent Profile Group
