# Supplementary material for: Association of Childhood Social-Emotional Functioning Profiles at School Entry With Early-Onset Mental Health Conditions
Source: JAMA Netw Open. 2019 Jan 4;2(1):e186694. doi: 10.1001/jamanetworkopen.2018.6694 (PMC6324314; doi:10.1001/jamanetworkopen.2018.6694)
Supplement: Supplement. — eTable 1. Latent Profile Analysis Model Fit Comparison: Social-Emotional Functioning Among Kindergarten Children in British Columbia, Canada eTable 2. Standardized Mean Scores (and SDs) for Early Development Instrument Social-Emotional Subscales by Latent Profile Group [file jamanetwopen-2-e186694-s001.pdf]

Supplementary Online Content

Thomson KC, Richardson CG, Gadermann AM, Emerson SD, Shoveller J, Guhn M. Association of childhood social-emotional functioning profiles at school entry with early-onset mental health conditions. *JAMA Netw Open*. 2019;2(1):e186694. doi:10.1001/jamanetworkopen.2018.6694

eTable 1. Latent Profile Analysis Model Fit Comparison: Social-Emotional Functioning Among Kindergarten Children in British Columbia, Canada
eTable 2. Standardized Mean Scores (and SDs) for Early Development Instrument Social-Emotional Subscales by Latent Profile Group

This supplementary material has been provided by the authors to give readers additional information about their work.

© 2019 Thomson KC et al. *JAMA Network Open*.

eTable 1. Latent Profile Analysis Model Fit Comparison: Social-Emotional Functioning Among Kindergarten Children in British Columbia, Canada (N = 34,323)

Number of Latent Profiles	Log Likelihood Value	aBIC	Entropy	BLRT	Lowest Class Probability	Smallest Class Size	Smallest Class Proportion
1	-382412.71	764941.66	NA	NA	1	34323	1
2	-327988.96	656159.56	0.95	0.00	0.97	8307	0.24
3	-311943.74	624134.52	0.93	0.00	0.93	3081	0.09
4	-304171.75	608655.92	0.90	0.00	0.86	1824	0.05
5	-297795.87	595969.55	0.91	0.00	0.87	1506	0.04
6	-291595.94	583635.07	0.94	0.00	0.90	963	0.03
7	-288058.93	576626.46	0.94	0.00	0.89	581	0.02
8	-284930.45	570434.88	0.91	0.00	0.78	496	0.01
9	-281664.14	563967.65	0.93	0.00	0.82	467	0.01
10	-279379.12	559463.00	0.93	0.02	0.79	442	0.01

© 2019 Thomson KC et al. *JAMA Network Open.*

eTable 2. Standardized Mean Scores (and SDs) for Early Development Instrument Social-Emotional Subscales by Latent Profile Group (N = 34,323)

	EDI Social-Emotional Subscales[a]							
Latent Profile Group	Overall Social Competence	Responsibility & Respect	Approaches to Learning	Readiness to Explore	Prosocial & Helping	Anxious & Fearful[b]	Aggressive[b]	Hyperactive & Inattentive[b]
1	.58 (.58)	.60 (.25)	.60 (.43)	.45 (.31)	.45 (.83)	.30 (.69)	.42 (.29)	.52 (.42)
2	-.42 (.86)	.36 (.43)	-.15 (.76)	-1.79 (.70)	-.57 (.85)	-.64 (1.26)	.40 (.31)	.29 (.59)
3	-.47 (.71)	-.51 (.61)	-.55 (.71)	.25 (.47)	-.32 (.82)	-.11 (1.00)	-.10 (.62)	-.55 (.82)
4	-1.44 (.75)	-1.49 (.68)	-1.71 (.79)	-2.23 (.91)	-1.12 (.74)	-.83 (1.38)	-.38 (.82)	-.97 (1.01)
5	-1.00 (.70)	-1.56 (.56)	-.95 (.77)	.16 (.56)	-.74 (.80)	-.26 (1.05)	-1.91 (.96)	-1.40 (.94)
6	-2.08 (.62)	-2.87 (.76)	-2.31 (.76)	-1.29 (1.29)	-1.39 (.59)	-1.08 (1.35)	-3.31(1.31)	-2.54 (.79)

[a]For each item, teachers rated a student's behavior currently or within the past 6 months as "never or not true" (score of 0), "sometimes or somewhat true" (score 5), or "often or very true" (score of 10). "Don't know" was coded as missing. Scores for each subscale item were summed and divided by the number of items in the subscale to derive a subscale mean which was then z-standardized within the sample. Negatively worded items were reverse-coded for the anxious and fearful, aggressive, and hyperactive and inattentive subscales so that higher scores indicated better social-emotional functioning (used for the other positively worded subscales). This EDI scoring was developed in consultation with educators and community audiences without prior clinical or research background for ease of interpretation and dissemination of findings.
[b]Reverse coded.

© 2019 Thomson KC et al. *JAMA Network Open.*
